# Effect of Cuff Inflation on Blood Pressure, Arousals, Sleep Efficiency, and Desaturations: Sub-Analysis of the VAST Pilot Study

**DOI:** 10.3390/diagnostics13182874

**Published:** 2023-09-07

**Authors:** Thenral Socrates, Philipp Krisai, Andrea Meienberg, Michael Mayr, Thilo Burkard, Annina S. Vischer

**Affiliations:** 1 Medical Outpatient Department and Hypertension Clinic, ESH Hypertension Centre of Excellence, University Hospital Basel, 4031 Basel, Switzerland; andrea.meienberg@usb.ch (A.M.); michael.mayr@usb.ch (M.M.); thilo.burkard@usb.ch (T.B.); annina.vischer@usb.ch (A.S.V.); 2 Department of Cardiology and Cardiovascular Research Institute Basel, University Hospital Basel, University of Basel, 4031 Basel, Switzerland; philipp.krisai@usb.ch; 3 University of Basel, 4031 Basel, Switzerland

**Keywords:** arterial hypertension, sleep arousal, cuff-based blood pressure measurement, nocturnal hypertension, pulse transit time

## Abstract

The influence of cuff inflations on night-time measurements during 24 h ambulatory blood pressure (BP) measurements is unknown. We investigated the potential effect of cuff inflations on sleep parameters using measurements taken simultaneously with a cuffless device using pulse-transit-time (PTT). On the first day of measurement, standard cuff-based 24 h BP and cuffless measurements were simultaneously performed on the right and left arms (CUFF/PTT-D). In this experiment, 1–2 days after the first measurement, the cuffless device was worn alone (PTT-D). Only data from the cuffless device were analyzed. The following mean sleep parameters were analyzed: mean systolic and diastolic BP, arousals, sleep efficiency, total arousals, arousal per hour, and desaturations. In total, 21 individuals were prospectively enrolled. The mean (SD) age was 47 (±15) years, and 57% were female. The mean systolic asleep BP during CUFF/PTT-D and during PTT-D were 131 (±21) and 131 (±26) mmHg, respectively. The mean diastolic asleep BP values during CUFF/PTT-D and during PTT-D were 80 (±14) and 84 (±14) mmHg, respectively (*p* = 0.860, *p* = 0.100, respectively). Systolic and diastolic asleep mean difference was 0.1 (±18.0) and −3.6 (±9.8) mmHg, respectively. There were significantly more total arousals during PTT-D (*p* = 0.042). There were no significant differences seen in sleep efficiency (*p* = 0.339) or desaturations (*p* = 0.896) between the two measurement periods. We could not show any significant impact from cuff inflations during sleep, as documented by PTT-D measurements.

## 1. Introduction

Arterial hypertension is a well-known burden on global health care and the most significant risk factor that leads to vascular disease [1]. This systemic disease plays a critical role in around 8.5 million deaths yearly, caused by its negative effects, which manifest as strokes, ischemic heart disease, other vascular disease, and renal disease [2].

The PAMELA study demonstrated the value of ABPM (ambulatory blood pressure monitoring), as it provides night-time values and, therefore, a better assessment of cardiovascular risk [3]. Night-time measurements give a complete picture of blood pressure (BP) profiles and dipping and are the most important predictor of cardiovascular risk and long-term outcomes and, therefore, should be considered in terms of steering treatment [4,5,6,7]. Both home BP and ABPM are recommended to establish a diagnosis and monitor treatment [8]. Currently, ABPM has consistently proven to be the gold standard for blood pressure measurement because of its many advantages and has even been shown to be superior compared to office and home blood pressure measurements [9].

However, ABPM, when taken with standard cuff devices, is disruptive due to repeated inflations during sleep and could potentially influence the true representation of night-time BP profiles [10]. Not only is the disruption substantial to the patient’s compliance and willingness to repeat these tests in the future, but it has been implicated in potentially inaccurate measurements due to sympathetic arousal [11]. Data show that cuff inflations cause artefacts due to motor activity, cuff errors, arousals, and arrhythmias, and they affect measurement outcomes [10]. Cuff inflations have also been shown to specifically cause night-time arousals, which are directly proportional to beat-to-beat elevations in BP [11]. Historically, cuff-based measurements have been used to look at long-term cardiovascular outcomes and risks; however, even this method only indirectly measures blood pressure through pulse wave detection and maximum volume changes, which are prone to error [12,13].

Since 2015, a cuffless BP measurement device has been commercially available and in clinical use throughout Europe [14]. This device uses, after an initial contralateral cuff-based calibration measurement, pulse transit time (PTT) to estimate BP via an algorithm patented by the manufacturer. This medical device has been validated in accordance with the ESH International Protocol Revision 2010 for the Validation of Blood Pressure Measuring Devices in Adults (ESH IP 2010) [15]. This non-inflating measurement technique could potentially benefit patients and clinicians alike. According to the manufacturers, this would enable the acquisition of night-time BP values without sympathetic arousal. The comfort of the device could improve compliance and allow physicians to prescribe ABPM as needed [16]. Nevertheless, previous analysis has shown that the cuffless BP measurement devices measure higher night-time BP values compared to the cuff-based reference devices [17,18,19]. Thus, these new devices need to be used with caution.

The influence of the cuff on various night-time measurements is unknown and needs to be looked at further before trending toward cuffless devices. This is especially significant, as there may be an inherent bias in validation studies when comparing cuff-based measurements since the cuff inflations may lead to higher BP values when compared to measurements with only a cuffless device. The newly published 2023 ESH Guidelines for the management of arterial hypertension explicitly do not recommend cuffless devices for diagnosis or monitoring of BP [20]. This stresses the point that in clinical practice and research, we need to better understand if cuff-based devices are affecting BP measurements.

Therefore, the aim of this study was to gain further insight regarding the potential effect of cuff inflations on arousals, sleep efficiency, desaturations, and mean night-time BP.

## 2. Materials and Methods

### 2.1. Study Center

The study took place at the Hypertension Clinic of the Medical Outpatient Department of the University Hospital Basel, Basel, Switzerland. This hypertension clinic is an approved ESH (European Society of Hypertension) Center of Excellence [21].

### 2.2. Device Details and Familiarization

This non-invasive cuffless device was introduced at the Medical Outpatient and Hypertension Clinic at the University Hospital Basel in April 2015. At the time of enrollment, our clinic had two years of experience with the correct application of this device, with more than 500 measurements before the start of the study. Experienced cardiologists (TB, ASV), both ESH-certified hypertension specialists, read all device measurements.

The non-invasive cuffless medical device estimates BP based on the PTT technique and permits uninterrupted beat-to-beat BP monitoring. This device consists of a finger photoplethysmograph, three ECG leads, and a watch-like device with an integrated actigraph. The transit time of a pulse wave from the corresponding ECG R-wave to the finger photoplethysmography signal is calculated and used in the algorithm developed to determine the blood pressure measurement [18]. After a single cuff-based calibration measurement on the opposite upper arm, systolic and diastolic BP levels are calculated using a non-linear model incorporating changes in the PTT and its relation to BP [22]. Increased pulse wave propagation results in shorter PTT and is associated with higher BP and vice versa. Pulse wave propagation, and thus PTT, depends on arterial wall stiffness and tension, both of which vary according to BP differences [23,24]. An actigraph continuously measures activity or movement and is used to assess sleep disorders and, generally, the time a patient is asleep.

In the current study, we analyzed BP, arousals, sleep efficiency, and desaturation. Asleep BP cut-off values were adapted based on a previous publication from our research group, which listed J point values for detecting elevated BP using the cuffless device [18]. The following cut-offs for the detection of mean asleep systolic and diastolic hypertension with the cuffless device were ≥136 mmHg and ≥83 mmHg, respectively [18]. Arousals were defined as activity and position changes during the night. Sleep efficiency was defined as total sleep time (TST) divided by time in bed (TIB) taken from automatically generated reports [25]. TIB was defined as documented by the patient in self-reported patient protocols. Oxygen desaturations were defined as the number of desaturations during TIB with a minimum duration of 10 s and ≥4% oxygen desaturation and were then indexed to the time in bed [26].

The standard cuff-based device used on the first day of measurements was a Spacelabs 90217A (Spacelabs Healthcare, Snoqualmie, Washington, DC, USA) 24 h BP monitor [18].

### 2.3. Study Design

**Figure 1:**
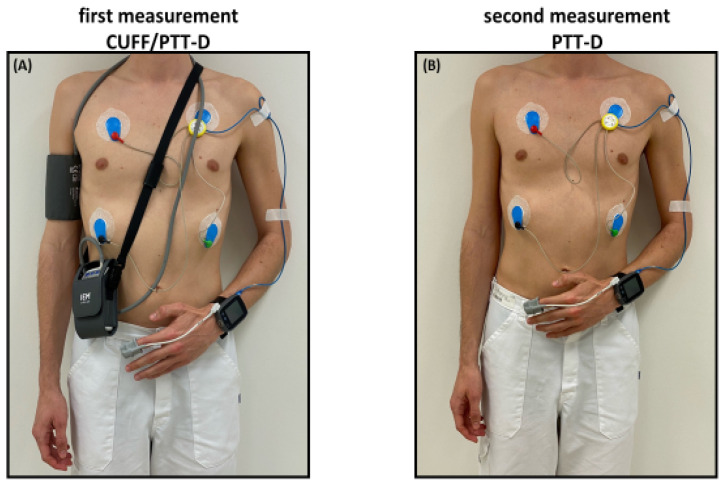
Study Flow: (**A**) first measurement with the cuffless device on the left arm and the reference device on the right arm (CUFF/PTT-D); (**B**) second measurement with only the cuffless device (PTT-D).

As reported previously, enrolment into the Somnotouch-NIBP Compared to Standard Ambulatory 24 h Blood Pressure Measurement, VAST Study (VAST = Validation Somnotouch, NCT03054688) took place at the Medical Outpatient and Hypertension Clinic at the University Hospital Basel between May and December 2017 [17,18]. VAST participants were a mixture of patients with an indication for a 24 h BP measurement and healthy volunteers. Inclusion criteria comprised the capacity to give informed consent and, at the time of enrollment, being at least 25 years of age. Exclusion criteria were younger than 25 years of age, a systolic upper arm blood pressure difference (right and left) of more than 10 mmHg, atrial fibrillation, and any reason not allowing for an upper arm blood pressure measurement. Both devices were mounted on the participant in a sitting upright position with legs uncrossed and back supported. An appropriately sized cuff was placed on the right arm and connected to the cuff-based ambulatory BP measurement device. The cuffless device was placed on the left forearm and connected to the photoplethysmograph on the left index finger. The ECG electrodes were placed according to the manufacturer’s instructions (Figure 1).

On the first day of measurement, a manually triggered cuff-based measurement was taken with the Spacelabs 90217A after 5 min of rest. This measurement was used as a calibration measurement for the cuffless device [27]. The cuff-based device was programmed for measurements every 20 min from 06:00 to 22:00 and every 30 min during the remaining period. Simultaneously, the cuffless device recorded beat-to-beat PTT according to the manufacturer’s standard programming. This part of the study is referred to as CUFF/PTT-D.

Participants of the VAST study were offered participation in the substudy and were consecutively enrolled [17]. As this substudy was initiated to generate a hypothesis regarding this topic, only 21 patients were enrolled. Consent was given for a second 24 h measurement with only the cuffless device within 4 days after the initial CUFF/PTT-D measurement. This substudy measurement is referred to as PTT-D. PTT-D only consisted of measurements taken with the cuffless device worn on the left arm and attached to the photoplethysmograph and ECG electrodes (Figure 1). For the second measurement, the cuffless device was similarly calibrated with a validated Omron 1300 device, as described above [28]. Participants were given questionnaires to individually record their activities, sleep schedules, medications, and biometrics.

### 2.4. Statistical Analysis

The distribution of continuous variables was determined using skewness, kurtosis, and a visual inspection of the histogram. The data were presented as medians (interquartile range) and means (±standard deviations). Comparisons were conducted using the paired Wilcoxon Signed Rank Test. Categorical variables were described as counts (percentages) and compared using Fisher’s exact test.

Statistical analysis was performed using R Version 4.2.3 with a *p*-value of < 0.05, which was pre-specified to indicate statistical significance [29].

### 2.5. Ethical Approval and Trial Registration

The VAST and substudy study protocol complies with the Declaration of Helsinki, was approved by the local ethics committee, Ethikkommission Nordwest und Zentralschweiz (Ethics Commission Northwest and Central Switzerland), (EKNZ 2017-00323), was registered (NCT 03054688) and externally monitored. All participants gave their informed consent.

## 3. Results

### 3.1. Baseline Characteristics

Table 1 shows baseline characteristics of the participants.

### 3.2. Blood Pressure Measurements

Table 2 outlines mean 24 h awake and asleep BP measurements on both days, with CUFF/PTT-D and PTT-D alone. CUFF/PTT-D had mean BP measurements of 134/83 mmHg, 139/86 mmHg, and 131/80 mmHg, respectively. PTT-D had mean BP measurements of 136/88 mmHg, 140/91 mmHg, and 131/84 mmHg, respectively. PTT-D had higher means during all phases of BP measurement. However, there was no significant difference between the groups, except for diastolic 24 h (*p* = 0.023) and diastolic awake (*p* = 0.019) measurements.

### 3.3. Asleep Hypertensive versus Normotensive Classification Differences between CUFF/PTT-D and PTT-D. Formatting of Mathematical Components

*Asleep Systolic Hypertension:*

**Figure 2:**
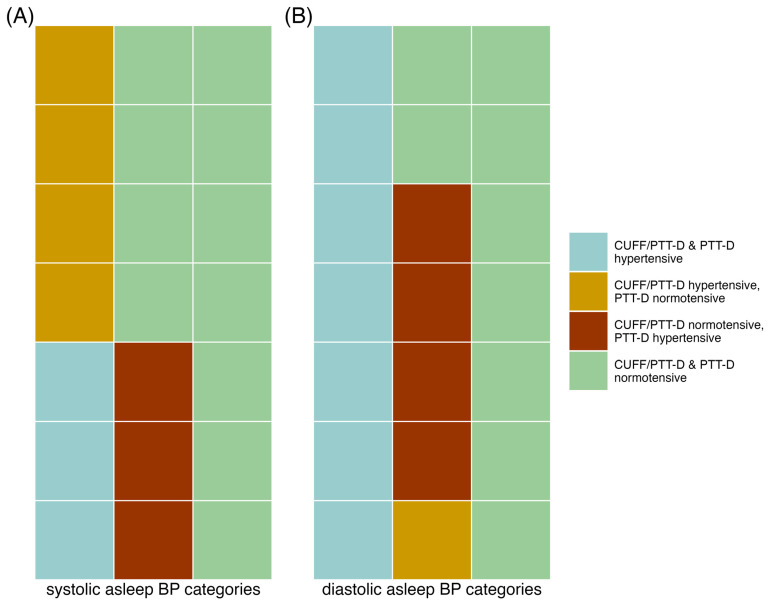
Tile graphs showing the various hypertensive classifications of patients according to each device; each tile represents one individual patient. (**A**) mean systolic asleep hypertensive classification; (**B**) mean diastolic asleep hypertensive classification.

Hypertensive values with CUFF/PTT-D and PTT-D were seen in three (14%) of the patients. Hypertensive values with CUFF/PTT-D and normotensive values with PTT-D were seen in four (19%). Normotensive values with CUFF/PTT-D and hypertensive PTT-D values were seen in three (14%). Normotensive values were seen in 11 (52%) with both the CUFF/PTT-D and PTT-D. Seven (33%) had discrepancies during the two measurement periods with mean asleep systolic hypertensive categorizations (*p* = 0.820) (see also Figure 2A).

*Asleep Diastolic Hypertension:*

Hypertensive values with CUFF/PTT-D and PTT-D were seen in seven (33%) of the patients. Hypertensive with CUFF/PTT-D and normotensive with PTT-D was found in one patient (5%). Normotensive with CUFF/PTT-D and hypertensive with PTT-D were seen in four (19%). Normotensive measurements in both were seen in nine (43%). Discrepancies with asleep diastolic hypertensive categorizations were seen in five (24%) (*p* = 0.310) (see also Figure 2B).

### 3.4. Arousal as a Potential Effect of Cuff-Based Measurements

**Figure 3:**
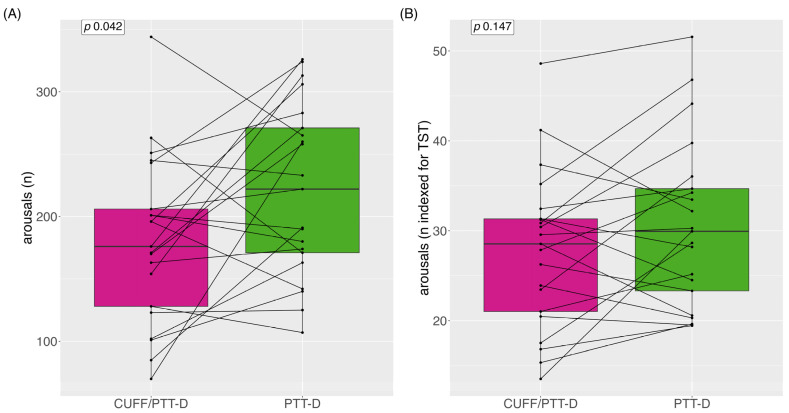
Arousals shown depicted as boxplots; (**A**) totals of arousals; (**B**) arousals index for total sleep time. Data are shown as medians and interquartile range, with each line representing one individual patient.

The total median (IQR = interquartile range) number of arousals for CUFF/PTT-D and PTT-D at night were 222 (110) and 176 (99), respectively, (*p* = 0.042) (Figure 3A). The number of CUFF/PTT-D and PTT-D total mean (SD) arousals at night were 221 (±67) and 180 (±65), respectively. Median arousals indexed during the total sleep time (TST) (in hours) were 29 (11) and 30 (13) for CUFF/PTT-D and PTT-D, respectively (*p* = 0.147) (Figure 3B).

**Figure 4:**
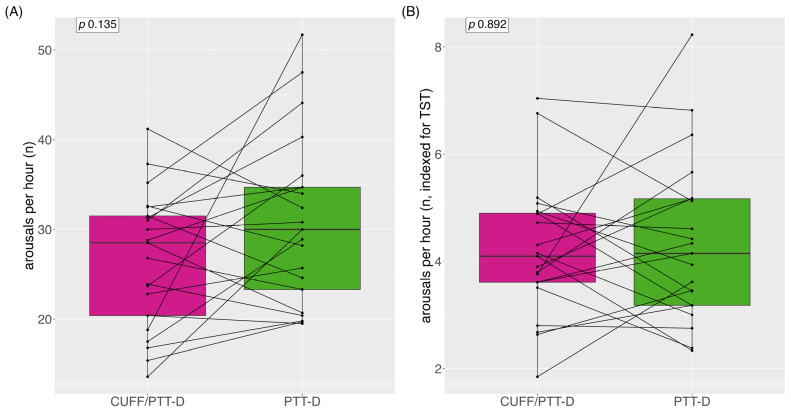
(**A**) arousal per hour; (**B**) arousals per hour indexed for total sleep time. Data are shown as medians and interquartile range, with each line representing one individual patient.

Median (IQR) arousals per hour with CUFF/PTT-D and PTT-D were 29 (12) and 30 (13) (*p* = 0.135), respectively (Figure 4A). Mean (SD) arousals per hour with CUFF/PTT-D and PTT-D were 27 (±7) and 31 (±9) (*p* = 0.135), respectively. Increased arousals with PTT-D alone were shown in 14 patients (66%) (Figure 4A).

Median (IQR) arousals per hour indexed to TST were 4 (1) and 4 (2) for CUFF/PTT-D and PTT-D, respectively (*p* = 0.892) (Figure 4B).

### 3.5. Sleep Efficiency

**Figure 5:**
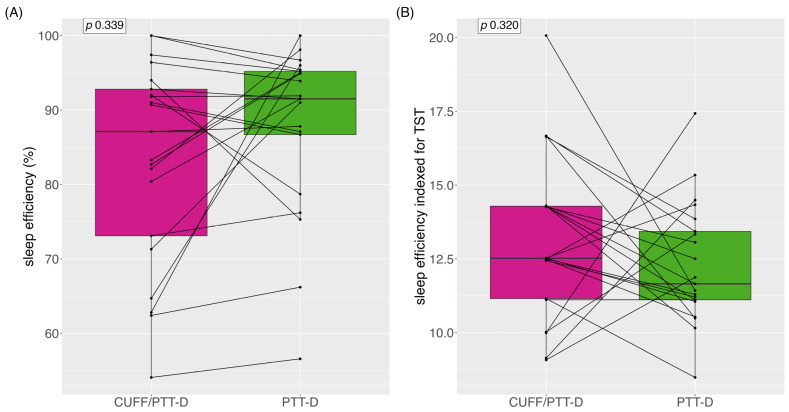
Sleep efficiency depicted as boxplots: (**A**) sleep efficiency with CUFF/PTT-D and PTT-D; (**B**) sleep efficiency indexed for total sleep time. Data are shown as medians and interquartile range, with each line representing one individual patient.

Median (IQR) sleep efficiency with CUFF/PTT-D and PTT-D were 87.1% (21) and 91.5% (12.6) (*p* = 0.339), respectively (Figure 5A). Mean (SD) sleep efficiency with CUFF/PTT-D and PTT-D were 83% (±13.6) and 87.9% (±11), respectively. Nine (43%) patients showed reduced sleep efficiency with PTT-D alone (Figure 5)

Median (IQR) sleep efficiency indexed during the total sleep time (TST) (in hours) was 12.5% (3.2) and 11.7% (2.6) for CUFF/PTT-D and PTT-D, respectively (*p* = 0.320) (Figure 5B).

### 3.6. Desaturation Index

**Figure 6:**
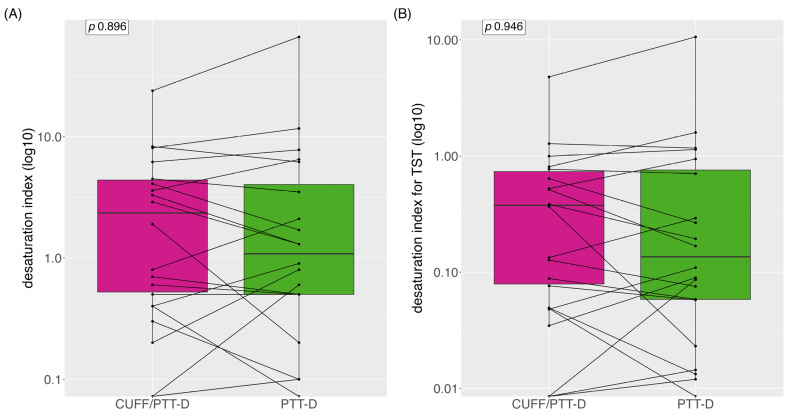
Desaturations depicted as boxplots as median desaturation; (**A**) desaturation with CUFF/PTT-D and PTT-D; (**B**) desaturation with CUFF/PTT-D and PTT-D indexed for total sleep time. Data are shown as medians (log) and interquartile range, with each line representing one individual patient.

Median (IQR) desaturation index was 0.8 (3.95) and 0.9 (4.5) with CUFF/PTT-D and PTT-D, respectively; no significant difference was seen between the groups (*p* = 0.896) (Figure 6A). The mean (SD) desaturation index was 3.4 (±5.4) and 5.4 (±14.4) with CUFF/PTT-D and PTT-D, respectively. When indexed with TST median (IQR), the desaturation index was 0.13 (0.65) and 11.65 (2.6) for CUFF/PTT-D and PTT-D, respectively (*p* = 0.95) (Figure 6B).

### 3.7. Mean and Mean Differences of Sleep Parameters during the Two Measurement Days

The mean (SD) differences regarding sleep parameters between the two measurement days are outlined in Table 3. A significant difference in total sleep time was seen, with the mean CUFF/PTT-D and PTT-D being 389 (±82) and 435 (±64), *p* = 0.048.

## 4. Discussion

To our knowledge, this is the first study that looks at the possible impact of cuff-based inflations on night-time BP measurements. The cuff inflations had no impact on the mean systolic BP, but a possible impact on the mean diastolic BP when looking at the mean differences. However, there was no statistically significant difference in terms of the mean systolic asleep and the mean diastolic asleep values during CUFF/PTT-D measurements compared to during the PTT-D measurements. We showed that when classifying the two measurement periods of each individual patient into hypertensive or normotensive, there were more mean asleep systolic hypertensive classifications with simultaneous measurements of CUFF/PTT-D than with PTT-D alone. In this small cohort, we could demonstrate significantly more arousals during the PTT-D alone than during CUFF/PTT-D; however, this difference was not statistically significant if indexed to the TST.

Increasingly, data suggest that nocturnal measurements obtained via ABPM are a stronger predictor of cardiovascular outcomes than awake ambulatory measurements [30]. However, convincing patients to undergo 24 h ABPM is a challenge. Cuff-based measurements and their inflations cannot be easily ignored and are reported as being disruptive. Within hypertension circles, there are often debates regarding how cuff-based measurements influence sleep quality and, thus, nocturnal or asleep BP values [31,32]. In our cohort, we saw no unequivocal influence of cuff inflations on mean asleep cuffless device measurements. We were able to document these parameters using readings taken simultaneously with a cuffless device during two separate days of measurement. However, this hypothesis could not be confirmed, especially when using cut-off values to differentiate between mean normotensive or hypertensive.

In the past few decades, new ways to measure BP have become available and easily implementable. However, the question remains if we should continue to only use cuff-based measurements or if other measurement modalities could reduce the potential influence of cuff inflations. New methods for measuring BP without repeated cuff inflations are interesting for clinicians and patients alike. Techniques using PTT seem to be a promising step in the right direction because of their comfort and increased number of measurements during the testing period. On the other hand, data and recent publications have shown that PTT devices are not directly comparable in terms of measuring blood pressure and result in higher BP values when validated against a gold standard cuff-based 24 h blood pressure measurement [18,19]. Additionally, indirect vs. direct measurement and beat-to-beat vs. mean blood values are not interchangeable clinically because outcome studies have never used cuffless blood pressure measurement data [3,5,20].

The results of the current analysis show that there are no significant differences between the night-time BP values when both devices are worn together vs. only one device. The mean asleep systolic blood pressure measurements were the same during both measurement days, and diastolic values were higher during the day when only wearing the PTT-D. It was found that 33% had discrepancies in their classification of nocturnal systolic hypertension and 24% of nocturnal diastolic hypertension classification. The cuffless device cut-off for nocturnal diastolic hypertension was 83 mmHg, based on our previous publication, which was also our mean value during the second day of measurement, causing more reclassifications [22]. This could have potential therapeutic consequences.

Interestingly, 66% of patients had more arousals when only wearing the cuffless device. This needs to be studied further. A possible explanation is that patients do not reach the sleep depth/stages for arousals to be detected by the cuffless device when wearing both devices. In addition, it has been reported that sleep time or time in bed was shorter during the first set of measurements when wearing both devices. This could mean that the absolute number of arousals is not the correct comparison. We, therefore, indexed the arousals to the TIB and per-hour total sleep time. After this, the number of arousals was still higher in the CUFF/PTT-D phase; however, it did not reach statistical significance. Sleep efficiency, the ratio of TST/TIB, in general, varies based on age. According to the National Sleep Foundation, ≥85% sleep efficiency is considered an indicator of good sleep quality; however, many publications use the cut-off ≥80% [33,34]. Although sleep efficiency was above 80% in both groups, it was 88% during the PTT-D measurements compared to 83% during the CUFF/PTT-D phase, demonstrating that cuff inflations may have an impact on this sleep parameter.

According to the manufacturer’s compendium, the cuffless device has the advantage of less sleep disturbances [25]. We did not find this to be the case. Our data indicate that the presumption that cuff-based devices may have caused arousals, sleep disturbances and eventually higher mean BP may be incorrect, especially in terms of mean BP values.

## 5. Strengths and Limitations

The strength of this study was the measurement of BP over a 24 h period on two closely spaced days. First, we used two separate devices simultaneously to record measurements and then with only one device for comparison on the same person. We believe that we addressed a crucial but understudied aspect of cuff inflations and their potential influence on night-time blood pressure measurements.

The main limitation of this analysis was the small number of patients. As this substudy of the VAST study was intended to generate a hypothesis, we did not include more patients. However, it is possible that this created a potential selection bias, as the participants would have had to be willing to have a second measurement taken with only the cuffless device. In addition, two different calibration devices were used on the two days of measurements. Moreover, there were possible discrepancies between the two days when the measurements were taken, especially in terms of activity, stress, and other possible influences on blood pressure. We used mean asleep values, which although clinically important, do not elucidate the effect of individual cuff inflations on corresponding BP measurements. Most importantly, although this analysis was restricted to night-time readings, we know, according to total sleep time, that both asleep periods were different. In contrast to other cuff-based vs. cuffless comparison studies, we did not have access to the additional Y-connection device to compare simultaneously measured cuff-based measurements to beat-to-beat cuffless measurements [10]. Therefore, we could only use mean values.

## 6. Conclusions

Cuff inflations did not seem to influence all the analyzed night-time sleep parameters and mean BP values but might lead to the reclassification of night-time hypertension.

## Figures and Tables

**Table 1:** Baseline Characteristics.

Characteristic	Overall (n = 21)
Female sex (%), n	12 (57%)
Age, years (±SD)	47 (±14.5)
BMI, kg/m^2^ (±SD)	25 (±4.4)
Antihypertensive treatment, n (%)	5 (23.8%)

Data represented as mean (±standard deviation) or counts (percentage).

**Table 2:** Mean blood pressure measurements.

Blood Pressure Measurement Period	CUFF/PTT-D BP	PTT-D BP	Mean Difference	*p*-Values
24 h systolic	134 (21.5)	136 (25.4)	−2.2 (18.0)	0.689
24 h diastolic	83 (13.9)	88 (13.7)	−5.4 (9.5)	0.023
awake systolic	139 (22.0)	140 (26.0)	−2.1 (16.7)	0.945
awake diastolic	86 (13.8)	91 (13.5)	−5.5 (9.2)	0.019
asleep systolic	131 (21.3)	131 (25.8)	0.1 (18.0)	0.860
asleep diastolic	80 (14.0)	84 (14.2)	−3.6 (9.8)	0.100

Data presented as mean (±standard deviation) and *p*-values; BP blood pressure; BP expressed as mmHg.

**Table 3:** Average and Mean Differences of Sleep Parameters.

Sleep Parameters	CUFF/PTT-D	PTT-D	Mean Difference	*p*-Values
Time in Bed, min	439 (±100)	472 (±71)	−32 (±132)	*p* = 0.259
Total Sleep Time (min)	389 (±82)	435 (±64)	−46 (±106)	*p* = 0.048
Arousals (events)	180 (±67)	221 (±69)	−40 (±78)	*p* = 0.400
Arousals Indexed to TST (events/TST)	27 (±8)	31 (±9)	−4 (±10)	*p* = 0.140
Sleep Efficiency %	83 (±14)	88 (±11)	−5 (±13)	*p* = 0.330
Sleep Efficiency Indexed to TST (%/TST)	14 (±3)	12 (±2)	1 (±4)	*p* = 0.305
Desaturation Indexed %	3 (±5)	5 (±41)	−2 (±9)	*p* = 0.930

Data are shown as mean (±standard deviation) and *p*-values.

## Data Availability

The datasets generated during and/or analyzed during the current study are not publicly available but are available from the corresponding author upon reasonable request.
